# Characterizing *Pv*ARP, a novel *Plasmodium vivax* antigen

**DOI:** 10.1186/1475-2875-12-165

**Published:** 2013-05-20

**Authors:** Darwin A Moreno-Pérez, Ambar Saldarriaga, Manuel A Patarroyo

**Affiliations:** 1 Fundación Instituto de Inmunología de Colombia (FIDIC), Carrera 50 No. 26-20, Bogotá, Colombia; 2 Universidad del Rosario, Calle 63D No. 24-31, Bogotá, Colombia

**Keywords:** *Plasmodium vivax*, Protein, Invasion, Antigenicity, Vaccine

## Abstract

**Background:**

*Plasmodium vivax* continues to be the most widely distributed malarial parasite species in tropical and sub-tropical areas, causing high morbidity indices around the world. Better understanding of the proteins used by the parasite during the invasion of red blood cells is required to obtain an effective vaccine against this disease. This study describes characterizing the *P. vivax* asparagine-rich protein (*Pv*ARP) and examines its antigenicity in natural infection.

**Methods:**

The target gene in the study was selected according to a previous *in silico* analysis using profile hidden Markov models which identified *P. vivax* proteins that play a possible role in invasion. Transcription of the *arp* gene in the *P. vivax* VCG-1 strain was here evaluated by RT-PCR. Specific human antibodies against *Pv*ARP were used to confirm protein expression by Western blot as well as its subcellular localization by immunofluorescence. Recognition of recombinant *Pv*ARP by sera from *P. vivax-*infected individuals was evaluated by ELISA.

**Results:**

VCG-1 strain *Pv*ARP is a 281-residue-long molecule, which is encoded by a single exon and has an N-terminal secretion signal, as well as a tandem repeat region. This protein is expressed in mature schizonts and is located on the surface of merozoites, having an apparent accumulation towards their apical pole. Sera from *P. vivax*-infected patients recognized the recombinant, thereby suggesting that this protein is targeted by the immune response during infection.

**Conclusions:**

This study showed the characterization of *Pv*ARP and its antigenicity. Further assays orientated towards evaluating this antigen’s functional importance during parasite invasion are being carried out.

## Background

Malaria is a tropical disease that causes millions of deaths per year around the world. The World Health Organization’s (WHO) Malaria Report 2011 indicated that there were 216 million cases and 655,000 deaths, mainly in children aged less than five years [1]. In spite of the incidence of cases worldwide and mortality index having become substantially reduced by 17% and 25% between 2000 and 2010, respectively, the figures regarding cases of malaria continue to be alarming. This is due to two main aspects impeding the total eradication of the disease: a gradual increase of parasite strains which are resistant to anti-malarial drugs [2] and populations of the mosquito vector which are insecticide-resistant [3].

*Plasmodium vivax* stands out as the most widespread parasite species causing malaria in humans; it is found throughout tropical and subtropical areas of the world and causes the disease’s highest morbidity indices on the Asian and American continents [4]. Even though it has been thought that *P. vivax* was a benign species, recent studies have shown that infection caused by this parasite could cause severe clinical symptoms [5,6], similar to those found in *Plasmodium falciparum* infection, thereby making it a potential menace.

Synthetic vaccines have been considered a good choice among control strategies when combating infectious diseases. Regarding malarial blood stages, vaccine development has been focused on the recombinant expression of parasite antigens (MSP-1 [7-9] and AMA-1 [10,11] having been the most studied) or on using synthetic peptides [12,13]; however, no fully effective vaccine against any species has been reported to date.

Recent work has established that the key to achieving an effective vaccine lies in blocking the interaction of parasite ligands which facilitate adhesion to target cell receptors [14]; this means that molecules localized on parasite surface and apical organelles (rhoptries and micronemes) must be identified. Unfortunately, data regarding the *P. vivax* proteins involved in invasion of reticulocytes that have been functionally characterized to date lag behind that available for their *P. falciparum* counterparts [15]. The foregoing has been due to the difficulty of standardizing an *in vitro* culture given poor reticulocyte recovery from adult human total blood [16]. Such experimental limitation has led to several study alternatives having been suggested; probabilistic techniques have been most useful when predicting possible vaccine candidates. A recent study involving hidden Markov models for analyzing the transcriptome of the *P. vivax* Sal-1 strain’s intra-erythrocyte life-cycle has led to the identification of 45 proteins that play a potential role in invasion; the role in cell adhesion for 13 of them (localized in merozoite rhoptries or on their surface) had previously been determined [17]. It was particularly interesting that an asparagine-rich protein (ARP) was found, this being conserved throughout the *Plasmodium* genus [17]. Only its *P. falciparum* orthologue has been described to date, called the apical asparagine-rich protein (*Pf*AARP) [18]. The *Pf*AARP-encoding gene has a prominent expression pattern towards the last intra-erythrocyte parasite development stage (48 hours post-invasion), which has been shown by real-time PCR and Northern blot. Antigenicity assays have shown that the N-terminal protein’s region (*Pf*AARP-N) obtained as a recombinant is recognized by antibodies from patients who have been naturally infected by *P. falciparum.* Rabbit antibodies directed against *Pf*AARP-N have been able to significantly inhibit parasite invasion of RBC *in vitro*. The foregoing, together with an RBC binding assay involving the expression of the complete protein on COS cell surface, has highlighted this antigen’s functional role in parasite binding to and invasion of target cells [18].

The present study was thus aimed at characterizing the asparagine-rich protein orthologue for *Pf*AARP in *P. vivax*. Molecular biology assays and immunochemistry techniques were used to demonstrate *Pvarp* gene transcription, protein expression and localization, as well as the ability to induce an antigenic response in patients who had suffered episodes of *P. vivax* malaria.

## Methods

### Selecting the gene and designing the primers and synthetic peptides

*Pv*ARP was selected, bearing in mind the *in silico* study by Restrepo-Montoya *et al.*[17] of *P. vivax* proteins playing a potential role in invasion. The PlasmoDB [19] database was then scanned to obtain the *Pvarp* gene sequence from the Salvador 1 (Sal-1) reference strain and to analyze adjacent genes’ synteny in different *Plasmodium* species. Specific primers were designed manually using Gene Runner software (version 3.05). B-cell lineal epitopes were predicted with AntheProt software [20] using the deduced amino-acid (aa) sequence. A tBlastn analysis of the predicted B-cell epitopes was then carried out to select peptide sequences exclusive for the *P. vivax* ARP.

### Animal handling

The experimental animals used were handled in accordance with Colombian Law 84/1989 and resolution 504/1996. *Aotus* monkeys kept at FIDIC’s primate station (Leticia, Amazon) were handled following established guidelines for the care and use of laboratory animals (National Institute of Health, USA) under the constant supervision of a primatologist. All experimental procedures involving *Aotus* monkeys had been previously approved by the Fundación Instituto de Inmunología's ethics committee and were carried out in agreement with the conditions stipulated by CorpoAmazonia (resolution 00066, 13 September, 2006). An *Aotus* monkey was experimentally infected with the Vivax Colombia Guaviare 1 (VCG-1) strain and monitored daily to assess infection progress throughout the entire study (up to day 18) using Acridine Orange staining. The monkey was treated with paediatric doses of chloroquine (10 mg/kg on the first day and 7.5 mg/kg/day until the fifth day) and primaquine (0.25 mg/kg/day from the third to the fifth day) at the end of the study to guarantee parasite clearance from total blood. Once experiments were over, CorpoAmazonia officers supervised the primate’s return to its natural habitat in excellent health.

### Isolating the *Plasmodium vivax* parasite

VCG-1 strain parasites were maintained *in vivo* according to previously described methodology [21]. A *P. vivax*-infected blood sample (3 mL) was passed through a discontinuous Percoll gradient (GE Healthcare, Uppsala, Sweden) according to an already established protocol [22] for obtaining schizont-stage enriched parasite. The sample was then used as RNA, genomic DNA (gDNA) and total protein source.

### Extracting RNA and cDNA synthesis

Total RNA was extracted from the schizont-enriched sample using the Trizol method and treated with RQ1 (RNA-qualified) RNase-free DNase (Promega, Wisconsin, USA) according to the manufacturer’s recommendations. Complementary DNA (cDNA) was synthesized using a SuperScript III enzyme (RT+) (Invitrogen, California, USA) in the following conditions: 65°C for 5 min, 50°C for 1 hour and 70°C for 15 min. An additional reaction without the SuperScript III enzyme (RT-) was made for use as control. Following 15 min’ incubation at 37°C with RNase (Promega, USA) the product was stored at −70°C until its later use.

### Cloning, sequencing and bioinformatics analysis

The cDNA RT + and RT- samples, as well as the gDNA obtained using a DNA Wizard Genomic purification kit (Promega), were used as template in 10 μL PCR reactions containing 0.5 U/μL Accuzyme DNA polymerase (Bioline), 1x AccuBuffer, 2 mM MgCl_2_, 0.5 mM dNTP, 0.5 μM primers and DNAse-free water for completing the reaction volume. Specific primers were designed for amplifying a region containing the entire *Pvarp* gene (direct 5′- CATTTGATCAGAGACGAC -3′ and reverse 5′- TTGGCACTTTTGTCACGA -3′), or the encoding sequence without the signal peptide (direct 5′- atgTGCAACACAAATGGGAAAA -3′ and reverse 5′- CACGCCAAACAGCTTCA -3′); the protein expression start codon was included in the direct primer’s 5′ end. A set of primers which had been previously designed for amplifying the *Pvron1-a* region (direct 5′- atgGCGAAGGAGCCCAAGTG-3′ and reverse 5′- ATCCCTAGCAATGCTTCG -3′) [23] was used as control for cDNA contamination with gDNA. The PCR for the *Pvarp* gene began with a denaturing step at 95°C for 5 min, followed by 35 cycles at 95°C for 30 sec, 52°C for 10 sec and 72°C for 1 min. *Pvron1-a* PCR began with a denaturing step at 95°C for 5 min, followed by 35 cycles at 95°C for 30 sec, 56°C for 10 sec and 72°C for 1.5 min. A Wizard PCR preps kit (Promega) was used for purifying *Pvarp* gene amplicons obtained from independent PCRs done with the RT + sample, once quality had been evaluated by 1% agarose gel. Pure products were then ligated to the pEXP5 CT/TOPO expression vector and transformed in TOP10 *Escherichia coli* cells (Invitrogen). Various clones were grown to purify the plasmid, using an UltraClean mini plasmid prep purification kit (MO BIO laboratories, California, USA); insert integrity and its correct orientation were confirmed by sequencing using an ABI PRISM 310 genetic analyzer (PE Applied Biosystems, California, USA). VCG-1 strain *Pv*ARP was characterized *in silico* using SignalP 3.0 [24], FragAnchor [25], XSTREAM [26], tools and the Interpro database [27] to search for secretion signal or GPI-anchor sequences, tandem repeats and putative domains, respectively. Clustal W software was used for aligning genes and pertinent encoding sequences [28].

### Recombinant protein expression and purification

The pEXP5-*Pv*ARP recombinant plasmid which encodes the entire *Pv*ARP sequence without the signal peptide (confirmed by sequencing) was transformed in *E. coli* BL21-AI (Invitrogen), according to the manufacturer’s recommendations. A protocol described by Sivashanmugam and his group [29] with some modifications, was used for improving expression yield. Briefly, the cells were grown overnight at 37°C in 10 mL Luria Bertani (LB) medium containing 100 μg/mL ampicillin and 0.1% (w/v) D-glucose. The initial inoculum was then seeded in 100 mL LB volume with the same amount of the aforementioned ampicillin and D-glucose and left to grow at 37°C using ~300 rpm until reaching 0.5 OD_600_; 0.2% L-arabinose (w/v) was used for five hours to induce expression. The culture was spun at 13,000 rpm for 30 min and lysed in extraction buffer (EB) (6 M urea, 12 mM imidazole, 10 mM Tris-Cl, 100 mM NaH_2_PO_4_ and 10 mg/mL lysozyme) supplemented with protease inhibitors (1 mM PMSF, 1 mM iodoacetamide, 1 mM EDTA and 1 mg/mL leupeptin). *Pv*ARP recombinant expression (r*Pv*ARP) was verified by Western blot and the protein was then purified by solid-phase affinity chromatography using Ni^+2^-NTA resin (Qiagen, California, USA) following the manufacturer’s recommendations. Briefly, total lysate was incubated with the resin pre-equilibrated with EB overnight at 4°C. The r*Pv*ARP mixture coupled to the resin was placed on a column and then washed several times with EB to eliminate weakly bound proteins. The recombinant protein was eluted with EB containing imidazole at differing concentrations (20, 100, 250 and 500 mM) in 3 mL fractions, which were analyzed by Coomassie blue staining to verify the presence of a single band and then dialyzed in PBS, pH 7.0. A micro BCA protein assay kit (Thermo Scientific) was used for quantifying every fraction so obtained; a bovine serum albumin (BSA) curve was used as reference.

### Peptide synthesis and obtaining polyclonal antibodies

A 20 aa-long peptide (predicted to be a good B-cell epitope), located at the N-terminus of *Pv*ARP (CG- LDNLKAKESPSSNDDGVYAKG-GC), was synthesized according to a previously-established methodology [30], polymerized, lyophilized and characterized by RP-HPLC and MALDI-TOF MS. Five mg of peptide (called 38582 herein) were immobilized on a CNBr-activated Sepharose 4B column, according to the manufacturer’s recommendations. A pool of fifteen sera taken from patients who had suffered previous *P. vivax* malarial episodes (stored in FIDIC’s serum-bank, see the ‘Sample source’ section) was incubated with the peptide coupled to a Sepharose 4B column overnight at 4°C with constant shaking to purify specific antibodies against peptide 38582 (anti-*Pv*ARP_38582_). The retained antibodies were eluted with gradients of increasing salt concentration (50 mM-0.3 M NaCl); they were then dialyzed in PBS, pH 7.8, and stored at −20°C until use.

### SDS-PAGE and Western blot

Five μg r*Pv*ARP and 50 μg total parasite proteins were separated on 12% SDS-PAGE and then transferred to nitrocellulose membranes. After having been blocked with 5% skimmed milk in PBS-0.05% Tween for one hour, each membrane was cut into strips and individually analyzed as follows: strips with the recombinant protein were incubated for two hours at room temperature (RT) with anti-*Pv*ARP_38582_ serum fractions (1:100 dilution) in a solution of 5% skimmed milk in PBS-0.05% Tween to assess which of them contained anti-*Pv*ARP specific antibodies; one strip was incubated with an anti-histidine monoclonal antibody coupled to peroxidase (1:4,500) as positive control for Western blot. Serum fractions recognizing the recombinant protein were then used to detect *Pv*ARP in total parasite lysate in the aforementioned conditions. Once antibody reactivity had been eliminated by incubating anti-*Pv*ARP_38582_ serum with peptide 38582 for one hour at 37°C, then this solution was used as control. Following three washes with PBS-0.05% Tween (5 min per wash), the strips were incubated for one hour with phosphatase-conjugated goat anti-human IgG as secondary antibody (1:5,000) at RT. The blots were revealed with a VIP peroxidase (Vector Laboratories, Burlingame, Canada) or BCIP/NBT colour development substrate kits (Promega), according to the manufacturers’ indications.

### Indirect immunofluorescence assay (IFA)

*Plasmodium vivax*-parasitized reticulocytes were washed thrice with PBS and then diluted in this solution until obtaining five to seven schizonts per field evaluated by staining with Acridine orange. Twenty μL of the sample were fed per well on eight-well multitest glass slides (Biomedicals, Inc) and the supernatant was removed 10 min later. Once the samples were dry, they were fixed with 4% formaldehyde for 5 min at RT. Following five washes with PBS, the sample was incubated with 1% Triton X-100 for 5 min in the previously described conditions. After 10-min blocking at RT with 1% (*v/v*) skimmed milk in PBS, each sample was incubated for one hour at RT with anti-*Pv*ARP_38582_ antibodies (20 μL). The samples were then incubated with FITC-conjugated anti-human IgG antibody (Sigma) at 1:30 dilution for 45 min in the dark. The DNA was stained with DAPI (0.5 μg/mL) for 10 min at RT and the excess was removed by washing several times with PBS-0.05% Tween. Once the slides had been examined under an Olympus BX51 florescence microscope (using 100× oil immersion objective), Volocity software (version 5.3.2) was used for superimposing the images.

### Enzyme-linked immunosorbent assay (ELISA)

*Pv*ARP antigenicity was evaluated in triplicate using serum from patients who had been living in malaria-endemic areas in Colombia and had presented episodes of such infection. Sera taken from healthy individuals who had never suffered the disease were used as negative controls. Briefly, 96-well polysorb plates were covered with 1 μg/mL r*Pv*ARP overnight at 4°C and then incubated at 37°C for one hour. The dishes were blocked with 200 μL 5% skimmed milk - PBS-0.05% Tween for one hour at 37°C. Antibody reactivity against the recombinant protein was evaluated by incubating the plates with a 1:100 dilution of each human serum in 5% skimmed milk -PBS-0.05% Tween for one hour at 37°C. Following incubation of the dishes with peroxidase-coupled anti-human IgG secondary antibody (1:10,000) diluted in 5% skimmed milk - PBS-0.05% Tween for one hour at 37°C, a peroxidase substrate solution (KPL Laboratories, WA, USA) was added to reveal the reaction, according to the manufacturer’s recommendations. Optical density (OD) was detected at 620 nm with an MJ ELISA multiskan reader and then calculated by subtracting the OD value obtained from the control well (no antigen). A 0.11 cut-off value for evaluating the positivity threshold was determined by taking the average of the OD plus twice the standard deviation (2 ± SD) of healthy individuals’ sera reactivity.

### Statistical analysis

Differences in average OD for r*Pv*ARP recognition by *P. vivax*-infected patients’ sera and in the control group were evaluated using the Kruskal-Wallis rank-sum test. A 0.05 significance level was used for testing a stated hypothesis.

### Sample source

Sera were obtained from 38 patients who were living in malaria-endemic areas of Colombia and who had suffered previous episodes of *P. vivax* malaria (but not *P. falciparum*), as well as from 15 healthy individuals who had never been affected by the disease. All individuals signed an informed consent form after receiving detailed information regarding the study’s goals.

### Accession number

The nucleotide and aa sequences used here have been reported in the GenBank database, under accession number KC514070.

## Results and discussion

### Analyzing the *arp* gene in *Plasmodium* species

The *P. vivax* proteins identified as playing a potential role in invasion by profile hidden Markov models [17] led to *Pv*ARP being selected. According to the information provided by the PlasmoDB database, the *Pvarp* gene (access number: PVX_090210) was found to be located between base pairs 1,230,371 and 1,231,228 in chromosome 5 of the Sal-1 strain. Similar genes were also found in the genome of other *Plasmodium* species known to be causing malaria in humans (*P. falciparum* and *Plasmodium knowlesi*), apes (*Plasmodium cynomolgi*) and rodents (*Plasmodium berghei*, *Plasmodium yoelii* and *Plasmodium chabaudi*). When analyzing alignment, the *Pvarp* gene codified product was 61.19%, identical to its orthologue in *P. knowlesi* (PKH_052690), 53.15% to its orthologue in *P. cynomolgi* (PCYB_053680) and 33.68% to its orthologue in *P. falciparum* (PF3D7_0423400), while identity ranged from 23.61% to 22.22% regarding orthologues in *P. chabaudi* (PCHAS_052400), *P. yoelii* (PY06454) and *P. berghei* (PBANKA_052380). Such genes were located in a syntenic region, as corroborated by their open reading frame orientation and exon-intron structure. The foregoing supported the idea that the *Pvarp* gene has been derived from a common ancestor; however, experimental evidence concerning the functional role that the encoded protein might have in different parasite species remains to be determined.

### The *Pvarp* gene is transcribed in schizonts

**Figure 1:**
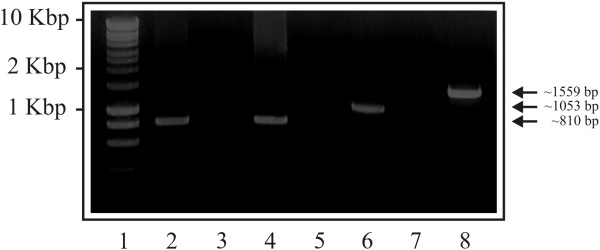
***Pvarp *gene transcription during blood stage.** Lane 1 indicates the molecular weight marker. Lanes 2–4 show *Pvarp* gene amplification using cDNA RT+, RT- and gDNA, respectively. Lanes 6–8 show the amplification of the *Pvron1-a* region using cDNA RT+, RT- and gDNA as template, respectively. Lane 5 shows the negative control for amplifying the *Pvarp* gene.

The presence of *Pvarp* gene transcripts in the *P. vivax* VCG-1 strain was confirmed by PCR using the cDNA from a parasite sample as template. Figure 1 shows the *Pvarp* gene amplification products (excluding the signal peptide-encoding region) (lanes 2–4) and the *Pvron1* gene’s *a* region (lanes 5–8) from cDNA and gDNA. A ~810 bp band (Figure 1; lane 2) obtained from cDNA amplification (RT+) showed that the *Pvarp* gene was transcribed in the schizont-enriched sample, similar to that reported in the transcriptional profile for the Sal-1 strain showing a maximum transcription level after 35 hours of intra-erythrocyte life cycle [31]. It was also confirmed that the *Pvarp* gene was encoded by a single exon once the sequences obtained from cDNA and gDNA products (Figure 1; lanes 2 and 4) had been aligned. The presence of a single ~1,053 bp band in *Pvron1-a* PCR (Figure 1; lane 6) indicated that the cDNA had not been contaminated by gDNA given that the expected product for the latter would have been ~1,559 bp (Figure 1; lane 8). No amplification was observed in the negative controls for each PCR (Figure 1; lanes 3 and 7 (RT-), and 5 (DNA-free water)).

Comparing *Aotus* monkey-adapted VCG-1 strain *Pvarp* gene sequences to those from the Sal-1 reference strain led to identifying four synonymous mutations, two non-synonymous ones producing aa changes (i e, methionine (M) for asparagine (N) and glycine (G) for N in aa position 217 and 219, respectively) and a 12-base pair deletion related to an asparagine-methionine-asparagine-glycine (NMNG) repeat block (Table 1). It has been found that parasite proteins have both highly polymorphic and conserved regions; the former are the target for an immune response while conserved sequences implicated in interaction with cell receptors are usually not antigenic [32]. Considering that the latter regions might be suitable targets for blocking parasite entry to host cells, further studies aimed at evaluating *Pvarp* gene polymorphism in different isolates are required to determine which sequences could be used as components of a vaccine against malaria caused by *P. vivax*.

**Table 1:** **Mutations found in VCG-1 strain *Pv*ARP nucleotide and amino acid sequences regarding the reference strain (Sal-1)**

Base pairs*	Amino acids*	**Mutations/Deletions**	**Changes in *Pvarp *nucleotide sequences in *P. vivax *strains**		**Changes in the *Pv*ARP amino acid sequences in *P. vivax *strains**	
			**Sal-I**	**VCG-1**	**Sal-I**	**VCG-1**
456	152	Synonymous	CAT	CAC	H	-
600-611	200-204	Deletion	CATGAACGGAAA	-	NMNG	-
650	216	Synonymous	TAT	TAA	N	-
651	217	Non-synonymous	GAA	CAA	M	N
655-656	218	Synonymous	CGG	CAA	N	-
657	219	Non-synonymous	AAA	CAA	G	N
663	221	Synonymous	AAC	AAT	N	-

*Nucleotide and amino acid positions are numbered according to *Pv*ARP in the Sal-I reference strain.

### Characterizing *Pv*ARP *in silico*

**Figure 2:**
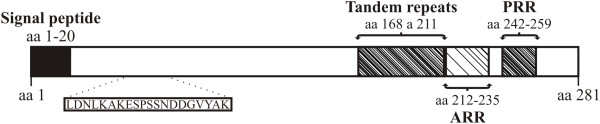
***In silico *characterization of *Pv*ARP, showing signal peptide localization, tandem repeats (TR), asparagine (ARR) and proline (PRR) amino acid repeat regions and the peptide selected for the antibody purification assay (shown in the box).**

The VCG-1 strain *Pvarp* gene encoded a 281 aa long protein having ~30 kDa molecular mass, this being 64 residues longer when compared to its homologue *Pf*AARP (217 aa) [18]. *Pv*ARP consists of 20% asparagine residues and has a signal peptide with a cleavage site between aa TNG-KS (Figure 2). A post-translational modification false positive consisting of a C-terminal glycosylphosphatidylinositol (GPI) anchor sequence has been predicted [17], differing to its *P. falciparum* homologue which has a true positive one. Asparagine- and proline-rich regions were found towards the C-terminal extreme of the protein sequence; the first of these covered residues 212 to 235, while the another one was found downstream between aa 242 and 259 (Figure 2). Additionally, a tandem repeat region (TR), a feature shared with other vaccine candidates described to date, was also found using XSTREAM software [26] (Figure 2); this region consisted of 11 repeat blocks from the (D/N/S)(V/M)NG consensus sequence found in aa 168 to 211. The sequence was seen to be exclusive for *P. vivax* and had mutations (two substitutions and four deletions), thereby suggesting that it was under pressure from the immune system. TR have been common in several *P. vivax* antigens described to date, which are mainly located on the surface or in apical organelles; these would include the circumsporozoite protein (CSP) [33], merozoite surface protein 9 (MSP-9) [34], *Pv*34 [35] and rhoptry neck proteins 1 and 2 [23,36]. Even though several studies have shown that the tandem repeats of *Pv*CSP trigger an immune response when inoculated in primates and humans [33,37,38], the response so produced did not completely inhibit infection caused by the parasite. It has been shown in other *Plasmodium* species that TR could act as a smokescreen against the immune system, thereby diverting strong reactions towards functionally-relevant regions [39]; however, their exact role in *P. vivax* antigens remains unknown.

### *Pv*ARP expression in schizonts and subcellular localization

**Figure 3:**
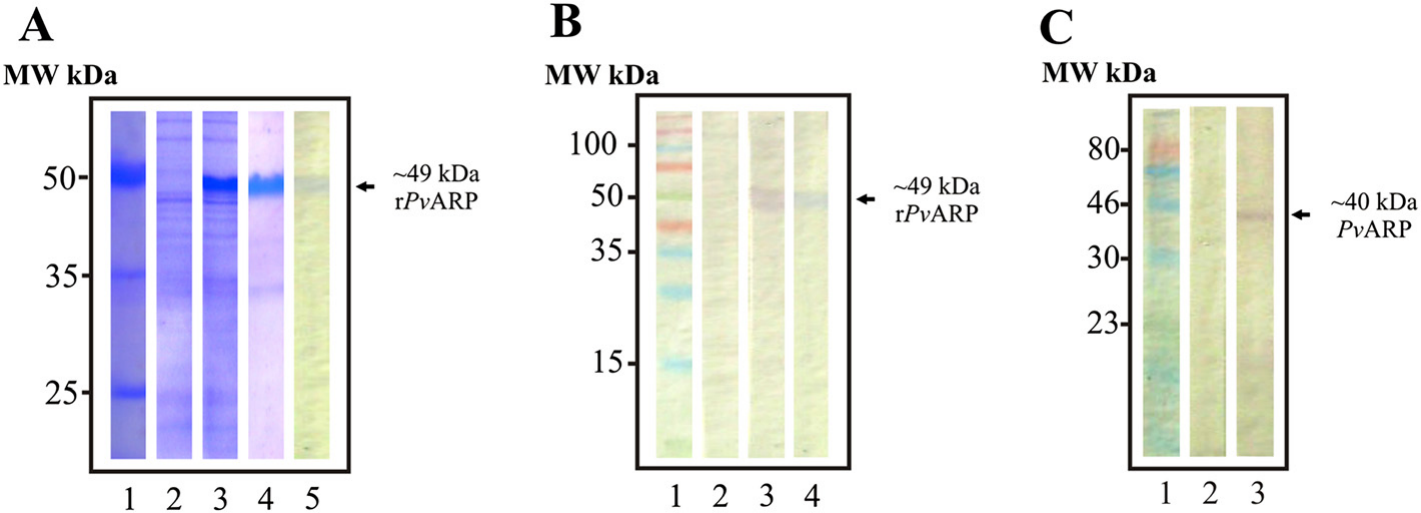
**Detecting recombinant and parasite protein by human antibodies.** (**A**) Recombinant protein expression and purification. Lanes 2–3 show non-induced and induced cell lysate, respectively (Coomassie staining). Lanes 4–5 show purified r*Pv*ARP stained with Coomassie or analyzed by Western blot using anti-polyhistidine antibodies, respectively. (**B** and **C**) Antibody ability to recognize recombinant and parasite *Pv*ARP by Western blot, respectively. Lane 2 shows the absence of human serum reactivity after being pre-incubated with peptide 38582. Lane 3 indicates *Pv*ARP recognition. Lane 4 shows detection of recombinant protein (positive control). MW kDa indicates molecular weight marker in kDa.

Specific human antibodies against an N-terminal *Pv*ARP synthetic peptide (Figure 2) were used for checking protein expression and localization in the schizont-enriched sample. *Pv*ARP was recombinantly expressed excluding the signal peptide and then purified (Figure 3A). Once human anti-*Pv*ARP_38582_ antibody ability to detect the recombinant protein in Western blot assays had been checked (Figure 3B), they were then used for detecting the protein on a blot containing parasite total lysate (Figure 3C). Both the parasite and recombinant *Pv*ARP proteins were detected above the expected weight (~40 and ~49 kDa, respectively), probably due to the presence of acidic aa (aspartic acid and glutamic acid) thereby causing anomalous migration on SDS-PAGE gel. The antibodies had specific reactivity to a ~40 kDa band; such reactivity was eliminated by using serum which had been pre-incubated with peptide 38582 (Figure 3C; lane 2).

**Figure 4:**
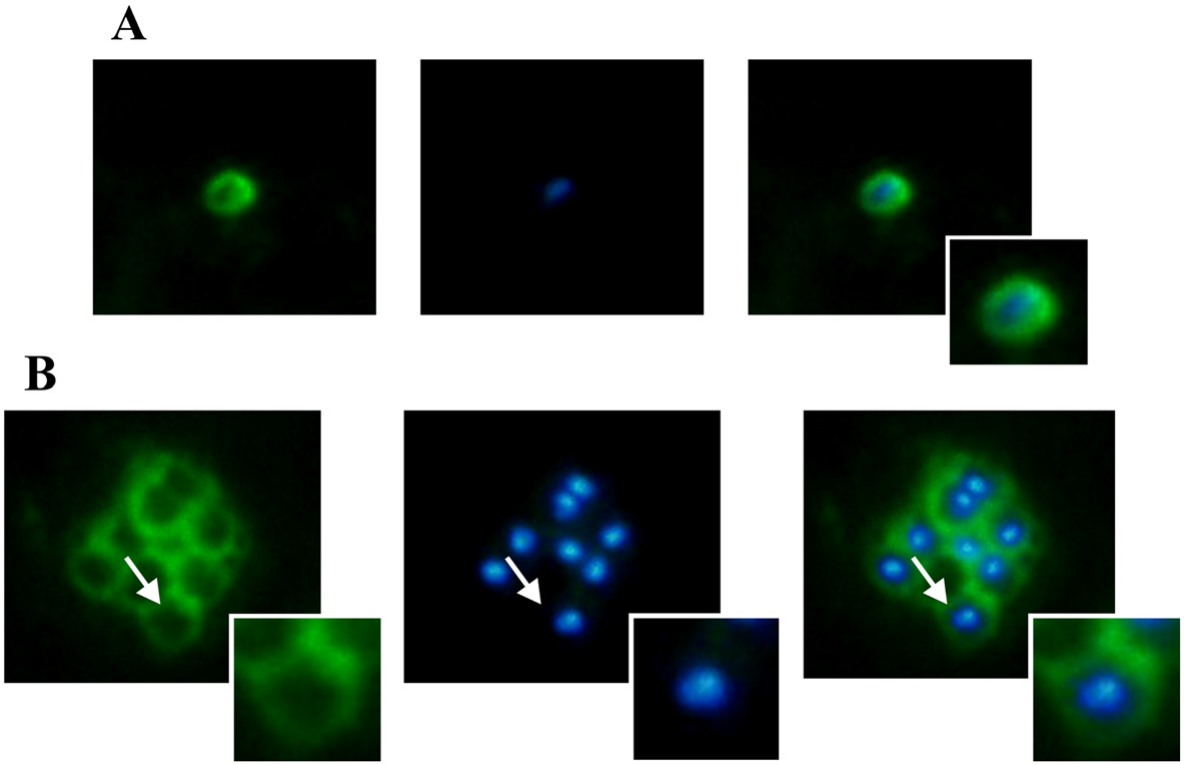
***Pv*ARP sub-cellular localization in mature schizonts.** (**A**) Shows the detection of the protein on free merozoite surface. (**B**) *Pv*ARP labelling on mature schizonts. The nuclei are labelled with DAPI (blue). An amplified image of a merozoite (indicated by an arrow) is shown in small boxes.

A strong fluorescence signal, having an apparent concentration towards the apical pole, was found on free merozoites’ surface and in mature schizonts when using the serum as primary antibody in the parasitized reticulocyte sample (Figure 4). The results led to the suggestion that *Pv*ARP could be expressed in apical organelles and then become relocated to the surface. However, other confocal or electron microscope assays are needed to determine the protein’s exact localization pattern.

### Antigenicity in humans

**Figure 5:**
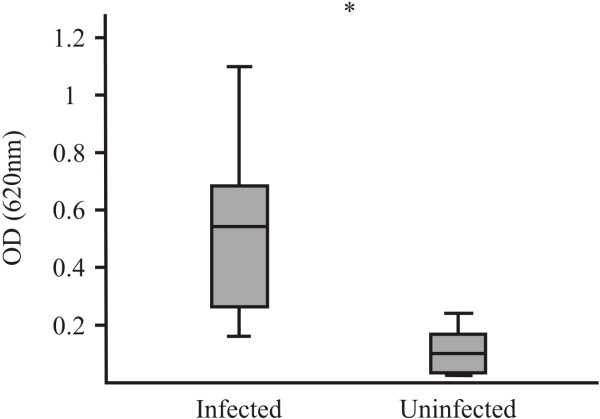
**r*Pv*ARP antigenicity.** The box diagram shows OD distribution (Y axis) for detecting r*Pv*ARP by sera from non-infected and infected individuals (X axis). *: Infected individuals (n = 38; X̄±S = 0.5 ± 0.2; 95%CI = 0.16-1.1) and control (n = 15; X̄±S = 0.1 ± 0.07; 95%IC = 0.03-0.24). p value = 0.000.

*Pv*ARP antigenic ability was evaluated by ELISA, using the sera from 38 patients who had suffered *P. vivax* malaria and 15 serum samples from people who had never suffered from the disease. The statistical test revealed a statistically significant difference between the medians (*m*) of the groups (Wilcoxon rank-sum test. Z = 5.1, p = 0.000); it gave *m* = 0.5 for the group of infected patients and *m* = 0.1 for the control group (Figure 5), thereby corroborating the fact that the protein was able to trigger an antibody response in the host during natural *P. vivax* malaria infection, most sera being able to recognize native and recombinant protein, as demonstrated by IFA and Western blot, respectively. The results supported the idea of analyzing this protein’s potential as a candidate for an anti-*P. vivax* vaccine.

## Conclusions

This study has described how the *P. vivax* asparagine-rich protein was characterized. As demonstrated, *Pv*ARP was conserved among different species belonging to the *Plasmodium* genus and shared some features of well-characterized surface and/or apical proteins being studied as candidates for a vaccine, such as prominent transcription and expression towards the end of the intra-erythrocyte life cycle and broad recognition by sera from patients infected with *P. vivax* malaria. The results supported the notion that this antigen could be a promising candidate for inclusion when developing an anti-malarial vaccine. Further immunogenicity assays and studies of the ability to induce protection in the experimental *Aotus* model are required.

## Competing interests

The authors declare that they have no competing interests.

## Authors’ contributions

DAMP designed experiments, analyzed data and wrote the initial manuscript. AS carried out molecular biology and immunochemical assays. MAP designed, evaluated and coordinated the assays and corrected the final manuscript. All authors read and approved the final manuscript.
